# Concurrent Infection of Hepatitis B Virus Negatively Affects the Clinical Outcome and Prognosis of Patients with Non-Hodgkin’s Lymphoma after Chemotherapy

**DOI:** 10.1371/journal.pone.0069400

**Published:** 2013-07-08

**Authors:** Jie Chen, Jianmin Wang, Jianmin Yang, Weiping Zhang, Xianmin Song, Li Chen

**Affiliations:** Department of Hematology, Changhai Hospital, Second Military Medical University, Shanghai, China; The University of Hong Kong, Hong Kong

## Abstract

Hepatitis B virus (HBV) is hepatotropic and lymphotropic. HBV-infected individuals have an increased risk of developing malignant lymphoma, and the HBV infection rate in lymphoma patients is significantly higher than that in the general population. However, the exact mechanism and correlation between HBV infection and lymphoma onset and progression currently remain unclear. We retrospectively analyzed clinical data from non-Hodgkin’s lymphoma (NHL) patients with different HBV infection statuses. The results showed that the HBV infection rate was significantly higher in patients with B-cell type and late stage of NHL. The chemotherapy efficacy for NHL patients with chronic active HBV infection was significantly lower than that for the patients with chronic inactive HBV infection, the patients with HBV carriers and the patients without HBV infection. In addition, the NHL chemotherapy activated HBV replication and caused significant liver dysfunction, which could further reduce the chemotherapy efficacy. Through Kaplan-Meier survival curve and log-rank analysis, we found that the HBV infection status in NHL patients was significantly correlated with the patients’ progression-free survival (PFS) and overall survival (OS). Compared with the patients without HBV infection (PFS: 95% CI 47.915 to 55.640; OS: 95% CI 81.324 to 86.858), the PFS and OS of the patients with chronic active HBV infection were significantly shorter (PFS: 95% CI 9.424 to 42.589, *P* < 0.001; OS: 95% CI 42.840 to 82.259, *P* = 0.006). The study demonstrated that the sustained HBV replication in patients with chronic active HBV infection could be a key factor that influences the prognosis of NHL patients after chemotherapy, and thus may provide information for designing rational clinical treatments for NHL patients with different HBV infection statuses and improve the treatment efficacy and prognosis.

## Introduction

China is a high endemic area for the hepatitis B virus (HBV). HBV is a DNA virus with both hepatotropic and lymphotropic characteristics [1–3]. The HBV-infected individuals have an increased risk of developing malignant lymphoma, and the HBV infection rate in lymphoma patients is significantly higher than that in the general population and in patients with other diseases. These findings indicate that HBV infection and lymphoma are mutually influential and interrelated [4,5]. Large-scale epidemiological investigations revealed that HBV could be a risk factor for B-cell lymphoma, particularly diffuse large B-cell lymphoma [6,7]. The HBV-infected individuals’ risk of developing non-Hodgkin’s lymphoma (NHL) is 2- to 3-fold greater than the risk of general population, but HBV infection is not related to Hodgkin’s lymphoma (HD) or other types of lymphoma [8,9]. The patients with HBV infection often develop lymphoma at an early age and at more advanced clinical stages [10]. The general rate of HBV infection is low in developed countries, but significantly higher in patients with malignant lymphoma compared with the general population [11]. The positive rate of hepatitis B surface antigen (HBsAg) in the general population in Italy is only 2.8%, whereas 8.5% in B-cell lymphoma patients [12]. The HBV infection rate is 1.2% in the Japanese healthy blood donor population but 7.3% in NHL patients [13]. This infection rate is as high as 30.8% in Romanian NHL patients, and this rate is significantly higher than the 6.3% infection rate in the general population [14]. In the NHL patients in Singapore, the HBV infection rate is 10.3% and significantly higher than the 4.1% infection rate in the general population [15]. Moreover, an early study in China also discovered that the general population had an HBsAg-positive rate of 7.18%, whereas the HBV infection rate in NHL patients was higher as 23.5% [16].

The exact mechanism through which HBV infection causes NHL remains unclear. Because HBV is a hepatotropic and lymphotropic DNA virus, it can infect lymphoid tissue, bone marrow hematopoietic cells, and peripheral blood mononuclear cells to replicate [1,17]. HBV-DNA, particularly the X and C genes, can frequently be detected in the blood mononuclear cells of NHL patients with HBV infection. The HBV-X gene product promotes the upregulation of epidermal growth factor receptor (EGFR) expression, which results in the malignant monoclonal proliferation of lymphocytes and ultimately leads to the development of malignant lymphoma through the binding site for nuclear factor kappa-light-chain-enhancer of activated B cells (NF-κB) on B-lymphoma cells and human T-cell leukemia cells [18,19]. HBV can also exert direct tumorigenic effects by stimulating the HBV-X protein to trans-activate genes that are associated with the control of the cell growth and the suppression of p53 function, thus initiating and maintaining HBV carcinogenesis [11]. After HBV infects NHL patients, the cellular and humoral immune abnormalities in these patients cause impaired viral clearance, which further stimulates the pathological changes and tumor progression observed after infection [20]. Furthermore, the comprehensive treatment for lymphoma, which is based mainly on chemotherapy, may stimulate HBV reactivation in NHL patients. These patients frequently develop severe liver dysfunction or liver failure that can endanger their lives, thus seriously affecting their treatment efficacy, prognosis, and survival longevity [21,22].

Thus, HBV infection and NHL onset and progression have a significant correlation. However, the effect of HBV on the incidence, treatment efficacy, and prognosis of lymphoma patients and the effect of lymphoma chemotherapy on HBV status remain unclear. Further studies are needed to clarify these questions. We retrospectively analyzed the clinical data from lymphoma patients diagnosed in Changhai Hospital (Shanghai, China) over the past decade, determined their HBV status and investigated the correlation between HBV infection and lymphoma to obtain possible information for designing rational clinical treatments for lymphoma patients with different HBV infection statuses, and for improving the treatment efficacy and the patients’ prognosis.

## Materials and Methods

### Patients

This retrospective study was approved by the ethics committee of Second Military Medical University (Shanghai, China). A total of 929 NHL patients, who were treated in the Department of Hematology, Changhai Hospital (Shanghai, China) from December 2000 to December 2010 and whose HBV infection statuses were examined, were included in this study (Table 1). The subjects included 598 male and 331 female patients. The male-to-female ratio was 1.8:1. The subjects’ median age was 52 years (range: 11 to 85 years). All of the lymphoma patients’ diagnoses were classified and confirmed by histopathological examination. The diagnosis, classification, and staging were determined according to the World Health Organization (WHO) Classification of Tumors, Pathology and Genetics of Tumors of Hematopoietic and Lymphoid Tissues in 2001 and the Ann Arbor Staging Criteria. All of the patients signed the informed consent form, and the written consent was obtained from the next of kin, caretakers, or guardians on the behalf of the minors/children participants before their information was stored in the hospital database and used for research purposes.

**Table 1:** Histopathological classifications of NHL.^*^

**B-cell lymphoma**		**n**	**T-cell lymphoma**		**n**
Diffuse large B cell lymphoma		341	Peripheral T-cell lymphoma-NOS		55
B-cell prolymphocytic leukemia		60	NK/T-cell lymphoma		40
Mucosa-associated lymphoid tissue lymphoma		57	T-lymphoblastic lymphoma		35
Follicular lymphoma		40	Anaplastic large cell lymphoma		27
B-cell chronic lymphocytic leukemia		32	Angioimmunoblastic T cell lymphoma		24
Mantle cell lymphoma		27	T-cell lymphocytic leukemia		16
B-cell lymphoblastic lymphoma		15	Hepatosplenic γδT-cell lymphoma		5
Splenic marginal zone lymphoma		10	Subcutaneous panniculitis-like T cell lymphoma		4
Lymphoplasmacytic lymphoma		7	Cutaneous mycosis fungoides		1
Burkitt lymphoma		5			
**Total**		**594**	**Total**		**207**

* Another 128 patients with an unclear classification of NHL were not included.

### Clinical Treatment

It was difficult to judge the curative effect for the patients who were administered one to two courses of chemotherapy. Therefore, only the patients who were treated with 3 or more than 3 courses of chemotherapy regimens were included in the investigation of chemotherapeutic outcome evaluation. Of the 929 NHL patients, most patients relapsed 2 to 5 times and received 3 to 8 courses of chemotherapy after every relapse, a total of 379 patients (262 of which were diagnosed with B-cell NHL) received 3 to 26 treatment courses (median: 12 courses) of CHOP chemotherapy regimen; 189 patients (all of which were diagnosed with B-cell NHL) received 3 to 15 treatment courses (median: 8 courses) of a rituximab-CHOP (RCHOP) regimen; 82 patients received 3 to 18 treatment courses (median: 8 courses) of CHOP-with-etoposide (CHOPE) regimen; 72 patients received 3 to 17 treatment courses (median: 6 courses) of FND regimen; 32 patients received 3 to 10 treatment courses (median: 5 courses) of the hyper-CVAD regimen; 26 patients received 3 to 13 treatment courses (median: 5 courses) of the VDCP regimen; and 23 patients did not receive any chemotherapy or immunotherapy treatment. After chemotherapy, the patients were subjected to the examinations of computed tomography scan, nuclear magnetic resonance imaging, ultrasound and biopsy.

The treatment efficacy was evaluated and classified according to the WHO criteria using the following categories: complete remission (CR, tumors disappeared completely and maintained for at least 4 weeks), partial remission (PR, the product of the largest tumor diameter and its vertical diameter was reduced by 50% or more, this condition was maintained for more than 4 weeks, and no new lesions appeared), stable disease (SD, the product of two vertical diameters was reduced by less than 50% or increased by less than 25%, and no new lesions appeared), and progressive disease (PD, the product of two vertical diameters was increased by more than 25%, or new lesions appeared). The patients did not receive any anti-virus agents during the period of NHL chemotherapy.

### Serum Liver Function Test

Peripheral blood was collected from all of the patients before and after chemotherapy. The liver function indicator levels, including alanine aminotransferase (ALT), aspartate aminotransferase (AST), gamma-glutamyl transpeptidase (GGT), albumin (ALB), total bilirubin (TBIL), and lactate dehydrogenase (LDH), were measured.

### HBV Detection

Enzyme-linked immunosorbent assay (ELISA) was used to determine the HBV infection status, including the hepatitis B surface antigen (HBsAg), hepatitis B surface antibody (HBsAb), hepatitis B core antibody (HBcAb), hepatitis B e antigen (HBeAg), and hepatitis B e antibody (HBeAb). Real-time quantitative polymerase chain reaction (RT-qPCR) was used to determine the HBV-DNA copy number before each chemotherapy cycle and two weeks after finishing chemotherapy. The HBV detection reagents were purchased from Shanghai Kehua Bio-engineering Co., Ltd (Shanghai, China). All of the patients were screened for the human immunodeficiency virus, hepatitis A virus, hepatitis C virus, hepatitis D virus, and hepatitis E virus to rule out the possibility of other viral infections.

### Follow-Up

Follow-up began at the time of diagnosis and ended on April 20, 2012. The median follow-up time was 26 months (range: 2 to 102 months). The progression-free survival (PFS) was calculated from the time of diagnosis to the time of relapse; for the patients without relapse, the time was calculated to the end of the follow-up period. The overall survival (OS) was calculated from the time of diagnosis to the end of follow-up period; for the patients who died, the time was calculated to the day of the patients’ death. For the patients who missed a follow-up visit during the period of study, the PFS and OS were calculated to the day of the last follow-up visit.

### Statistical Analysis

The significant differences in the baseline clinical parameters and treatment characteristics were evaluated by chi-square test and *t* test. The PFS and OS were calculated by Kaplan-Meier method and compared through the log-rank test. Two-tailed *P*<0.05 was considered statistically significant. The statistical analyses were performed with the PASW Statistics 18.0 software.

## Results

### Relationship between HBV Infection Status and NHL Clinicopathological Characteristics

To investigate the relationship between the HBV infection rate and the NHL clinicopathological characteristics (Table 2), the HBsAg-positive patients were used as the HBV infection group. The results showed that the HBV infection rate in NHL patients was 135/929 (14.53%). HBV infection was not correlated with gender (*P*=0.0841), age (*P*=0.9165), or histopathological classification (*P*=0.3151), but was correlated with cell type. Among the NHL lymphoma patients with HBV infection, the proportion of B-cell types was significantly higher than that of T-cell types [16.67% (99/594) vs. 10.14% (21/207), *P*=0.0450]. HBV infection was also correlated with the stage of lymphoma. The proportion of stages III and IV NHL patients with HBV infection was significantly higher than that of patients with stages I and II [16.08% (106/659) vs. 10.74% (29/270), *P*=0.0358; Table 2).

**Table 2:** Comparison of the clinical characteristics of HBsAg-positive and HBsAg-negative lymphoma patients.

**Clinical characteristics**			**n**	**HBsAg-Positive (n)**		**HBsAg-Negative (n)**		**χ^2^**	***P* value**
Age (NHL)								0.0035	0.9530
≥ 50 year			452	66		386			
< 50 year			477	69		408			
Gender (NHL)								2.5657	0.1092
Male			597	95		502			
Female			332	40		292			
Cell type (NHL) ^*^								5.1261	**0.0236**
B cell			594	99		495			
T cell			207	21		186			
Stage (NHL)								4.4044	**0.0358**
I–II			270	29		241			
III-IV			659	106		553			
Group (NHL)								1.0092	0.3151
Group A			450	60		390			
Group B			479	75		404			

* The patients with an unclear classification of NHL were not included.

### Relationship between HBV Infection Status and Chemotherapy Efficacy in Lymphoma Patients

**Figure 1:**
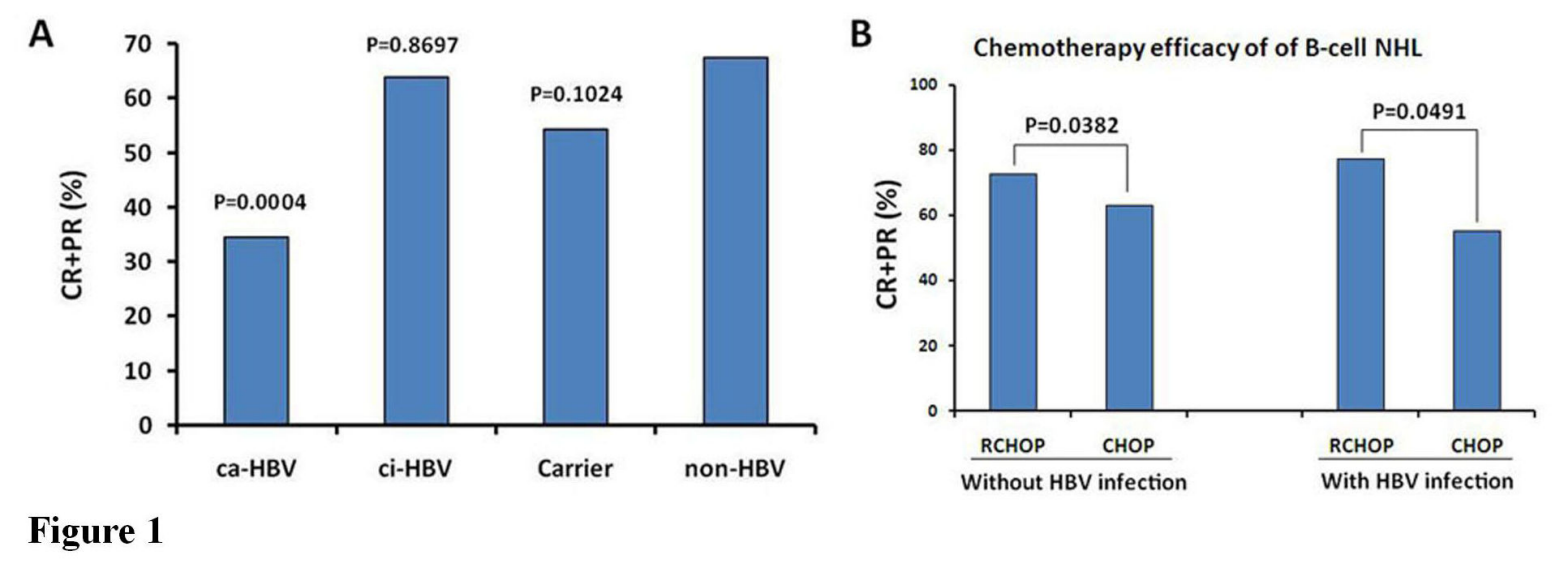
Relationship between HBV infection status and clinical outcome of chemotherapy in NHL patients. (**A**) The treatment efficacy was evaluated in 780 patients with NHL who received different types of chemotherapy regimens, and the relationship between the HBV infection status (ca-HBV, chronic active HBV infection; ci-HBV, chronic inactive HBV infection; Carrier, HBV carrier with HBsAg-positive; non-HBV, no HBV infection with HBsAg-negative) and the clinicopathological characteristics of lymphoma were analyzed by a chi-square test. (**B**) The analysis of the B-cell NHL patients who received RCHOP and CHOP chemotherapy regimens revealed that the effect of rituximab in chemotherapy was found to be more efficient in the groups without and with HBV infection.

Of the 929 NHL patients in this study, 906 received different types of chemotherapy regimens and 23 did not receive any treatment. To evaluate the chemotherapy efficacy, only the 780 patients who received 3 or more than 3 courses of treatments were included in the analysis and classified into the following groups: chronic active HBV infection (ca-HBV: HBsAg-, HBeAg- and HBcAb-positive), chronic inactive HBV infection (ci-HBV: HBsAg-, HBeAb- and HBcAb-positive), HBV carrier (Carrier: only HBsAg-positive), and no HBV infection (non-HBV: HBsAg-negative). We then investigated the relationship between the HBV infection status *in vivo* and the chemotherapy efficacy in lymphoma patients. The results revealed that the HBV infection status was closely correlated with the efficacy of chemotherapy in lymphoma patients (*P*=0.0020, Table 3). The ca-HBV group had a chemotherapy efficacy (CR + PR) of 9/26 (34.62%), which was significantly lower than the efficacy of 451/658 (68.54%) found in the non-HBV group (*P*=0.0004). In comparison, the treatment efficacy in the ci-HBV group (63.89%) and the HBV carrier group (47.06%) did not show significant differences from that of the non-HBV group (Figure 1A).

**Table 3:** Relationship between HBV infection status and chemotherapy efficacy in NHL patients.

	**n**	**ca-HBV**		**ci-HBV**		**Carrier**		**non-HBV**		**χ^2^**	***P* value**
Chemotherapy outcome										14.8142	**0.0020**
CR+PR	518	9		43		18		448			
SD+PD	262	17		21		15		209			
	**n**			**CR+PR**				**SD+PD**		**χ^2^**	***P* value** ^*^
HBV infection status											
ca-HBV	26			9				17		12.7328	**0.0004**
ci-HBV	64			43				21		0.0269	0.8697
Carrier	33			18				15		2.6677	0.1024
non-HBV	657			448				209			

Note: ca-HBV, chronic active HBV infection; ci-HBV, chronic inactive HBV infection; Carrier, HBV carrier (HBsAg-positive); non-HBV, no HBV infection;

* compared with the non-HBV group.

Of the 929 NHL patients, 189 B-cell NHL patients received the RCHOP chemotherapy regimen. To evaluate the effect of rituximab in chemotherapy, 262 B-cell NHL patients who received CHOP regimen were treated as the control group. Regardless of whether these patients had HBV infection, the effective rates of RCHOP were significantly higher than those of CHOP in the treatment of B-cell NHL (Figure 1B).

### Chemotherapy Effect on HBV-DNA Replication in NHL Patients

In the HBsAg-positive NHL patients, the HBV titer was determined in 73 patients before and after lymphoma chemotherapy. Before chemotherapy, the mean HBV titer was (8.15 ± 3.12) ×10^3^ copies/ml. After chemotherapy, the HBV titer increased in 61 cases and decreased in 12 cases. The mean HBV titer was (1.25 ± 0.52) × 10^4^ copies/ml. In general, the viral titer increased significantly after chemotherapy (*P*=0.0289).

### Effect of HBV Infection on the Liver Function of NHL Patients

**Figure 2:**
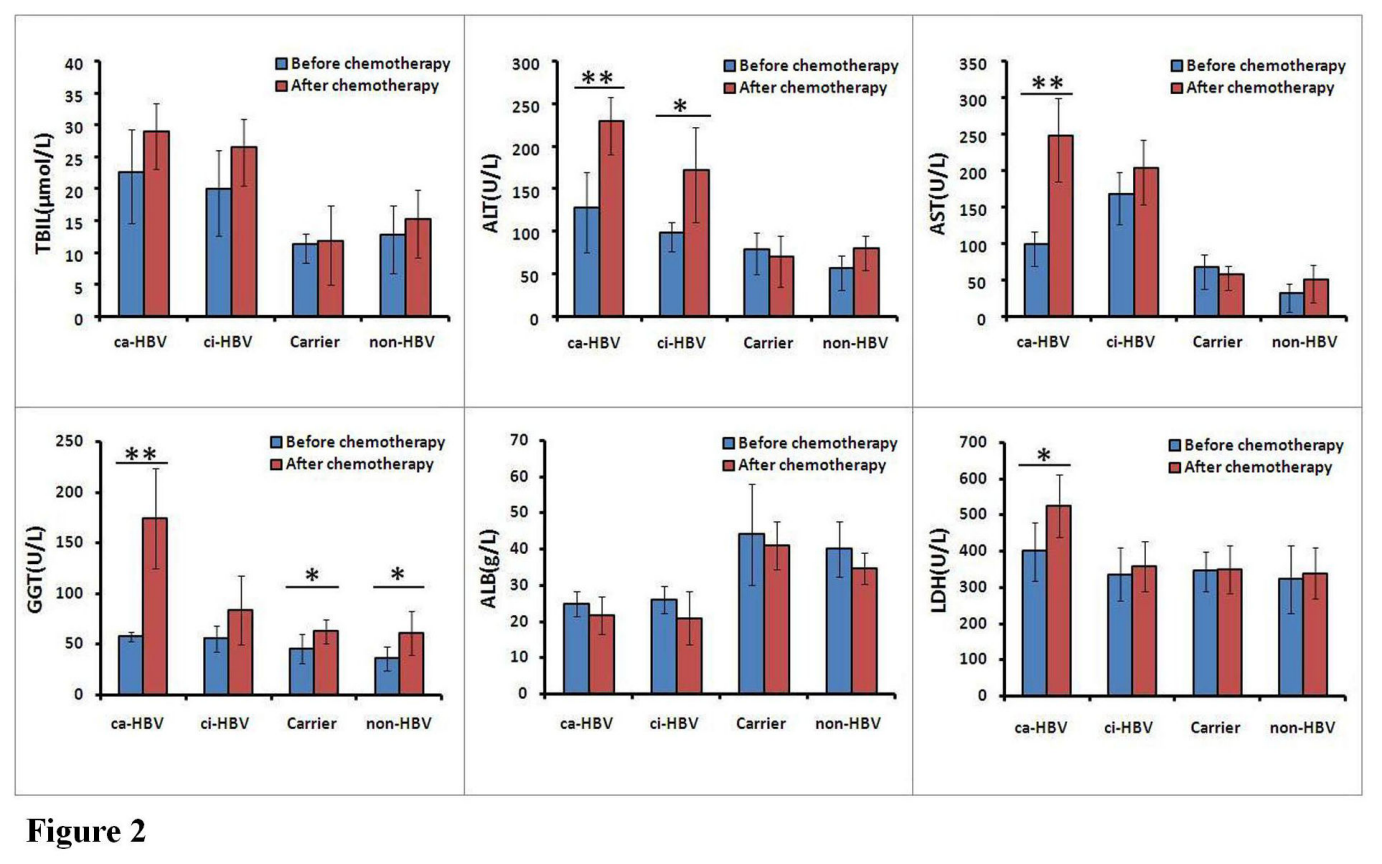
Effect of HBV infection status on the liver function of NHL patients. Peripheral blood was collected from 906 patients with NHL who received different types of chemotherapy regimens before and after chemotherapy, and the liver function indicator levels were measured. The data were expressed as the ‘mean ± standard deviation (SD)’ and statistically analyzed by Student’s *t* test; *P<0.05, **P<0.01. ca-HBV, chronic active HBV infection; ci-HBV, chronic inactive HBV infection; Carrier, HBV carrier with HBsAg-positive; non-HBV, no HBV infection with HBsAg-negative; TBIL, total bilirubin; ALT, alanine aminotransferase; AST, aspartate aminotransferase; GGT, gamma-glutamyl transpeptidase; ALB, albumin; LDH, lactate dehydrogenase.

The ALT and AST levels in 135 the HBV-infected patients were 101.44 ± 24.32 U/L and 111.00 ± 51.45 U/L, respectively, and these levels were significantly higher than the levels obtained in the 794 patients without HBV infection (ALT: 55.8 ± 20.04 U/L, *P*=0.0193; AST: 30.80 ± 19.49 U/L, *P*=0.0095). The HBV-infected patients exhibited variable increases in the TBIL, GGT, and LDH levels and slight decreases in the ALB levels compared with the patients without HBV infection; however, the statistical analysis did not show any significant differences (*P*>0.05). The liver function indicator levels after chemotherapy in the 906 patients who received chemotherapy revealed that, compared to the measures taken before chemotherapy, the ALT (228.67 ± 33.81 U/L vs. 127.17 ± 47.71 U/L, *P*=0.0017), AST (247.33 ± 57.53 U/L vs. 98.67 ± 23.76 U/L, *P*=0.0002), GGT (174.12 ± 0.39 U/L vs. 57.12 ± 5.03 U/L, *P*=0.0002), and LDH levels (524.72 ± 87.73 U/L vs. 398.98 ± 80.98 U/L, *P*=0.0274) in the patients with ca-HBV infection were all significantly increased. The ALT levels (171.00 ± 55.71 U/L vs. 98.33 ± 16.85 U/L, *P*=0.0121) of the patients with ci-HBV infection were increased; and the GGT levels in the HBV carriers (62.73 ± 11.91 U/L vs. 45.75 ± 14.34 U/L, *P*=0.0497) and the patients without HBV infection (60.60 ± 21.67 U/L vs. 35.57 ± 12.11 U/L, *P*=0.0331) were increased (Figure 2).

### Relationship between HBV Infection Status and NHL Patients’ Prognosis after Chemotherapy

**Figure 3:**
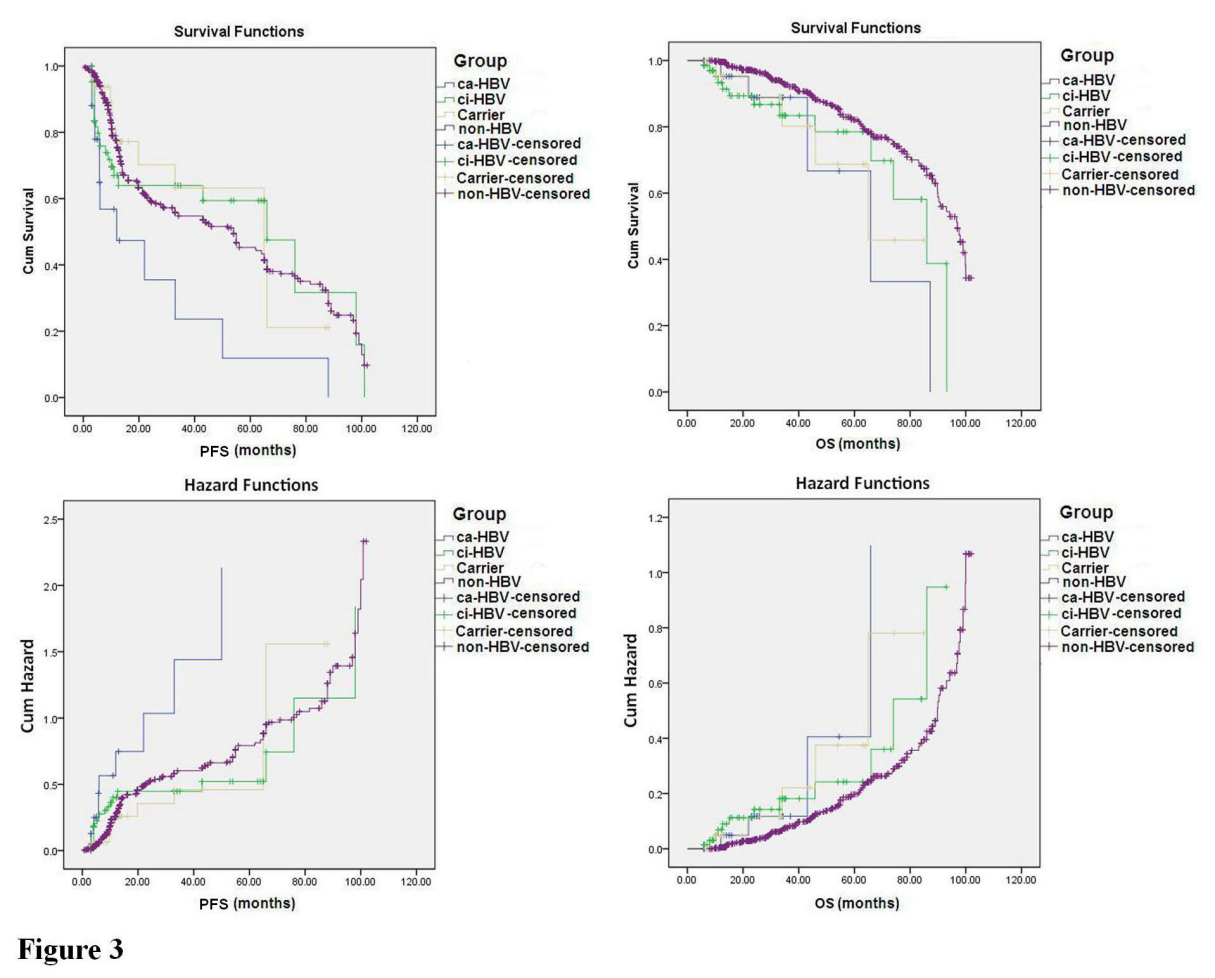
Survival and hazard functions of NHL patients with different HBV infection statuses after chemotherapy. The 780 NHL patients who received at least 3 courses of chemotherapy were followed-up, and the follow-up data were illustrated through Kaplan-Meier survival curves and analyzed by a log-rank test. The progression-free survival (PFS) time was calculated from the time of diagnosis to the time of relapse; for the patients without relapse, the time was calculated to the end of the follow-up period. The overall survival (OS) was calculated from the time of diagnosis to the end of the follow-up period; for the patients who died, the time was calculated to the day of the patient’s death. If the patients missed a follow-up visit in the middle of the study, the PFS and OS times were calculated to the day of the last follow-up visit. ca-HBV, chronic active HBV infection; ci-HBV, chronic inactive HBV infection; Carrier, HBV carrier with HBsAg-positive; non-HBV, no HBV infection with HBsAg-negative.

**Figure 4:**
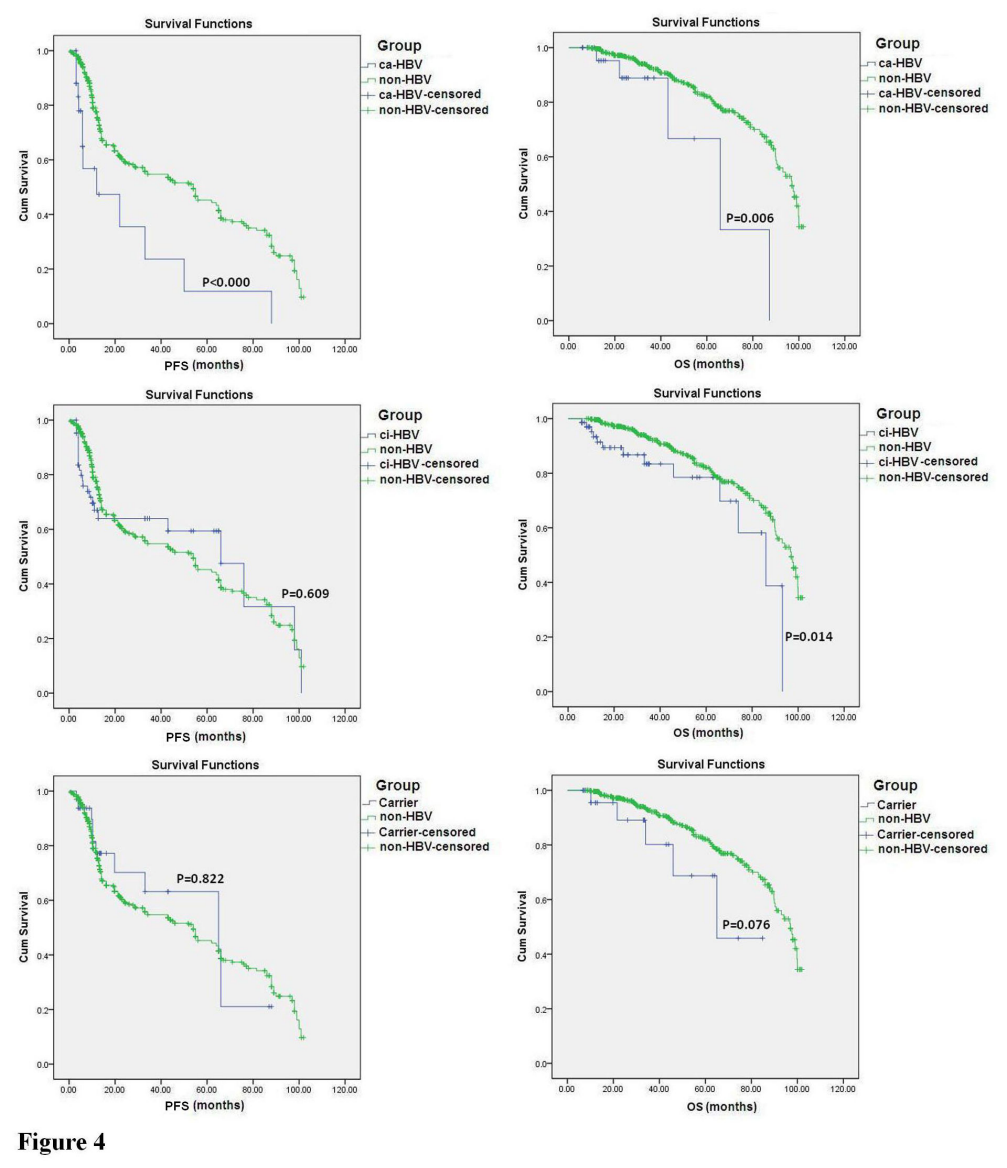
Comparison of different HBV infection statuses and non-HBV infection in NHL patients after chemotherapy. The follow-up data from 780 NHL patients who received at least 3 courses of chemotherapy were illustrated through Kaplan-Meier survival curves and analyzed by a log-rank test. Each group with a different HBV infection status was compared with the non-HBV infection group in NHL patients after chemotherapy.

In the study, the follow-up period for the 906 NHL patients who received chemotherapy began at the time of diagnosis and ended on April 20, 2012. The median follow-up time was 26 months (range: 2 to 102 months). To evaluate the NHL patients’ prognosis after chemotherapy, only the 780 patients who received 3 or more than 3 courses of treatment were included in the analysis. The Kaplan-Meier survival curve was plotted for the ca-HBV, ci-HBV, HBV carrier, and no HBV infection groups, and a log-rank analysis was performed. The results revealed that the PFS and OS differed significantly among the 4 groups with different HBV infection statuses [PFS: 95% confidence interval (CI) 47.865 to 54.993, *P*=0.015; OS: 95% CI 79.992 to 85.332, *P*=0.002], and the status of ca-HBV was the major risk factor for the survival of NHL patients (Figure 3). Compared to the non-HBV group (PFS: 95% CI 47.915 to 55.640; OS: 95% CI 81.324 to 86.858), the PFS and OS values for the ca-HBV group (PFS: 95% CI 9.424 to 42.589, *P*<0.001; OS: 95% CI 42.840 to 82.259, *P*=0.006) were significantly shorter. The OS (95% CI 62.229 to 82.322, *P*=0.014) was significantly shorter for the ci-HBV group, but the PFS was not (95% CI 41.709 to 69.543, *P*=0.609). The PFS (95% CI 38.382 to 65.186, *P*=0.822) and OS (95% CI 49.834 to 78.034, *P*=0.076) values for the HBV carrier group were not significantly different from those of the patients without HBV infection (Figure 4). These results indicated that the sustained HBV replication in patients with ca-HBV infection could be a key factor that influences the prognosis of NHL patients after chemotherapy.

**Figure 5:**
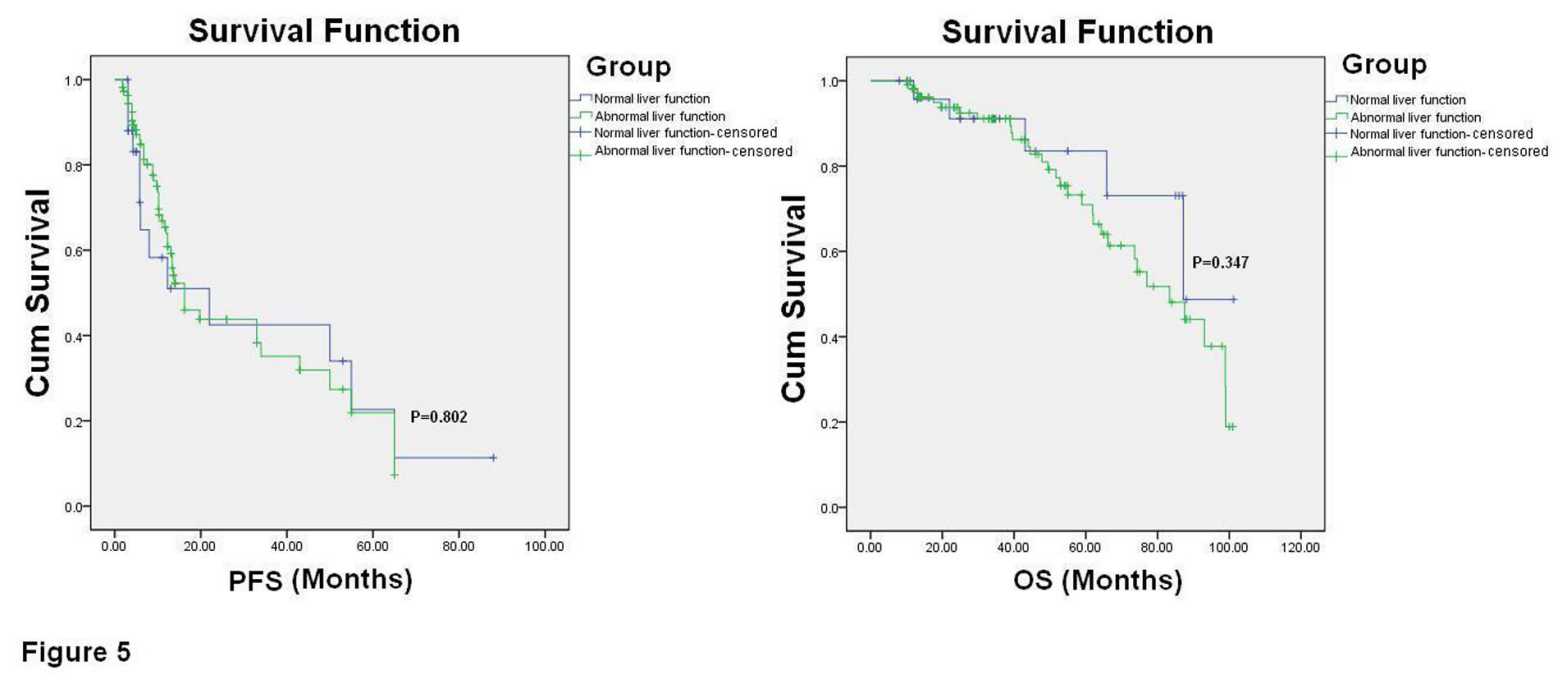
Influence of different liver function statuses before chemotherapy on the prognosis of NHL patients. The 135 HBV-infected patients were classified into two groups according to their ALT/AST levels before chemotherapy: the normal liver function group (n=26) and the abnormal liver function group (n=109). The OS and PFS are illustrated through Kaplan-Meier survival curves and analyzed by a log-rank test.

To demonstrate whether the HBV-mediated liver toxicity may have contributed to the worse outcome of NHL patients, the 135 HBV-infected patients were classified into two groups, namely the normal and abnormal liver function groups, according to the ALT/AST levels examined before chemotherapy. Although the OS and PFS were slightly shorter in the abnormal liver function group compared with the normal liver function group, but there were no significant differences for the OS and PFS between these two groups (OS: 95% CI 70.091 to 82.463, *P*=0.347; PFS: 95% CI 24.881 to 38.261, *P*=0.802; Figure 5).

## Discussion

HBV is not only hepatotropic, but it can also infect cells in lymphoid and hematopoietic tissue. Previous studies showed that the HBV infection rate in lymphoma patients is high, suggesting that HBV could be associated with lymphoma [23]. We collected 929 NHL patients over a 10-year period. The total HBV infection rate (HBsAg-positive) was 14.53% (135/929), which was higher than the reported 7.18% HBV infection rate in the general Chinese population and that of patients with other types of tumors [16,24]. Through a statistically analysis of the HBV infection status in our large scale of participants, we concluded that the HBV infection and the occurrence of 594 cases of B-cell NHL had a significant causal relationship and that HBV could be a relevant factor in the development of B-cell NHL. Moreover, the HBV-infected NHL group had a larger proportion of late-stage lymphoma (stages III and IV) patients, indicating that HBV infection could stimulate NHL progression, and this finding was consistent with the results reported in the literatures [10,25].

In China, HBV infection is a common complication in B-cell NHL. A higher prevalence of HBV infection was shown in patients with the B-cell subtype of NHL (30.2%), however, the HBV infection rate in patients with T-cell NHL was 19.8% [21]. Because HBV infection and replication can stimulate the NF-κB and p53-signaling pathways to promote the onset and development of lymphoma [11,18,19], the HBV infection status *in vivo* has serious effects on NHL treatment. Reports on the association between HBV infection and chemotherapy efficacy in NHL patients are sparse, and clinical studies with large sample sizes are also lacking. We statistically analyzed 780 NHL patients who received at least 3 courses of chemotherapy and found that the HBV status of the HBV-infected patients was correlated with the efficacy of chemotherapy. The chemotherapy efficacy in NHL patients with chronic active HBV infection was significantly lower than that found for patients with chronic inactive HBV infection, patients who were HBV carriers, and patients with no HBV infection. Because HBV is persistent and has a high replication status, we speculated that the constant stimulation from antigens in the HBsAg, HBeAg, and HBcAb triple-positive patients could further promote the onset and development of NHL, which would lead to reduced chemotherapy efficacy. Once the proliferation capacity and pathogenicity of HBV were weakened and the hosts developed a defensive state based mainly on antivirus antibodies, the chemotherapy efficacy for NHL could be improved. This result indicated that the role of HBV infection in the onset and development of NHL cannot be ignored and should be considered when designing a clinical treatment protocol. It is worth mentioning that, for the treatment of B-cell NHL, the effective rate of RCHOP was significantly higher than that of CHOP, suggesting that the use of rituximab in chemotherapy is desirable, regardless of whether the patients have HBV infection.

HBV infection and replication affect the efficacy of chemotherapy for the treatment of NHL, and NHL chemotherapy also affects the status of HBV replication [26,27]. Studies have revealed that HBsAg-positive patients with malignant lymphoma are at risk of HBV reactivation after chemotherapy. The HBV reactivation rate has been estimated to be 20% to 70% [4,28–30]. In patients who received rituximab combination therapy, the HBV reactivation rate was as high as 70%, and this resulted in a mortality of 13% [31–33]. The preventive application of lamivudine can reduce the risk of HBV reactivation and the mortality risk caused by HBV reactivation and may prolong the OS [15,34–36]. Of the 135 HBsAg-positive NHL patients in our study, the HBV titers were determined in 73 patients before and after chemotherapy. The results confirmed that the viral titer decreased in 12 cases and increased in 61 cases. The mean HBV titer markedly increased after chemotherapy. In addition, the high-level of HBV replication after chemotherapy caused significant liver dysfunction and significantly increased the ALT, AST, GGT, and LDH levels, which could further reduce chemotherapy efficacy.

In the NHL patients with HBV infection, the HBV replication affects the chemotherapy efficacy, and chemotherapy further activates HBV replication, which results in a poor prognosis [37]. We followed the 906 NHL patients who received different types of chemotherapy regimens. Using the Kaplan-Meier survival curve and log-rank analysis, we discovered that the HBV status of the NHL patients was significantly correlated with the patients’ PFS and OS. Compared with patients without HBV infection, the PFS and OS of the patients with chronic active HBV infection were significantly shorter. In the patients with chronic inactive HBV infection, only the PFS was significantly shorter, and the PFS and OS of the HBV carriers were not significantly different from those of patients without HBV infection. These results also demonstrated that the status of sustained HBV replication could be a key factor that influences the prognosis of NHL patients.

Our study revealed that it is necessary to determine the HBV status, HBV-DNA copy number, and liver function of NHL patients, particularly NHL patients with a history of hepatitis, during diagnosis and treatment. These measurements may provide important clinical information for the proper design of therapeutic regimens and the accurate evaluation of the treatment efficacy and prognosis [38]. For patients who have chronic active HBV infections in which the virus is in a state of high-level replication, anti-virus treatment prior to anti-NHL tumor treatment will inhibit HBV reactivation, thus reducing the liver dysfunction caused by chemotherapy and viral replication, and improving the chemotherapy efficacy and the patients’ prognosis.
